# DNA-free two-gene knockout in *Chlamydomonas reinhardtii* via CRISPR-Cas9 ribonucleoproteins

**DOI:** 10.1038/srep30620

**Published:** 2016-07-28

**Authors:** Kwangryul Baek, Duk Hyoung Kim, Jooyeon Jeong, Sang Jun Sim, Anastasios Melis, Jin-Soo Kim, EonSeon Jin, Sangsu Bae

**Affiliations:** 1 Department of Life Science, Hanyang University, Seoul, South Korea; 2 Department of Chemistry, Hanyang University, Seoul, South Korea; 3 Department of Chemical and Biological Engineering, Korea University, Seoul, South Korea; 4 Department of Plant and Microbial Biology, University of California, Berkeley, CA 94720-3102, USA; 5 Center for Genome Engineering, Institute for Basic Science, Seoul, South Korea; 6 Department of Chemistry, Seoul National University, Seoul, South Korea

## Abstract

Microalgae are versatile organisms capable of converting CO_2_, H_2_O, and sunlight into fuel and chemicals for domestic and industrial consumption. Thus, genetic modifications of microalgae for enhancing photosynthetic productivity, and biomass and bio-products generation are crucial for both academic and industrial applications. However, targeted mutagenesis in microalgae with CRISPR-Cas9 is limited. Here we report, a one-step transformation of *Chlamydomonas reinhardtii* by the DNA-free CRISPR-Cas9 method rather than plasmids that encode Cas9 and guide RNAs. Outcome was the sequential *CpFTSY* and *ZEP* two-gene knockout and the generation of a strain constitutively producing zeaxanthin and showing improved photosynthetic productivity.

Unicellular microalgae have enormous potential as cell factories for the generation of biomass, fuel-type molecules, and synthetic chemistry feedstock while, at the same time, helping to mitigate CO_2_ emissions in the atmosphere^1^,^2^. Thus, genetic manipulations of microalgae for increasing a feedstock of bioenergy, natural resources of biomedical and biochemical compounds generation highly attract attention from both academic and industrial establishments. For gene editing at desired target loci, programmable nucleases, such as zinc finger nucleases (ZFNs)^3^, transcription-activator-like effector nucleases (TALENs)^4^,^5^ and RNA-guided engineered nucleases (RGENs)^6^ derived from the type II CRISPR-Cas9 system have been applied to microalgae. However, although CRISPR RGENs are rapidly superseding ZFNs and TALENs due to the ease of application and low cost^7^, a recent study with them showed that stable expression of Cas9 in the model green microalga *Chlamydomonas reinhardtii* is cytotoxic, resulting in targeted mutagenesis at a frequency below 10^−9 ^6.

Recently, DNA-free RGENs, that is, preassembled Cas9 protein-gRNA ribonucleoproteins (RNPs), have enabled efficient genome editing in *C. elegans*^8^, human cells^9^, mice^10^ and plants^11^,^12^. The RGEN RNP delivery obviates the need for codon optimization or specific promoters in different species so that it can be conveniently and rapidly applied to various organisms. Furthermore, RGEN RNPs reduce off-target effects and mosaicism and may also be less cytotoxic in cells because the Cas9 protein is transiently active and then degraded by endogenous proteases in cells^9^. In addition, the resulting gene-edited plants and animals could be exempt from genetically modified organism (GMO) regulations due to the absence of foreign DNA sequences^13^. Here, we demonstrate targeted gene editing in *C. reinhardtii*, which has a fully sequenced and annotated genome and possesses haploid chromosomes^14^, by using DNA-free RGEN RNPs.

## Results and Discussion

### Targeted *CpFTSY* gene knockout using RGEN RNPs

**Figure 1:**
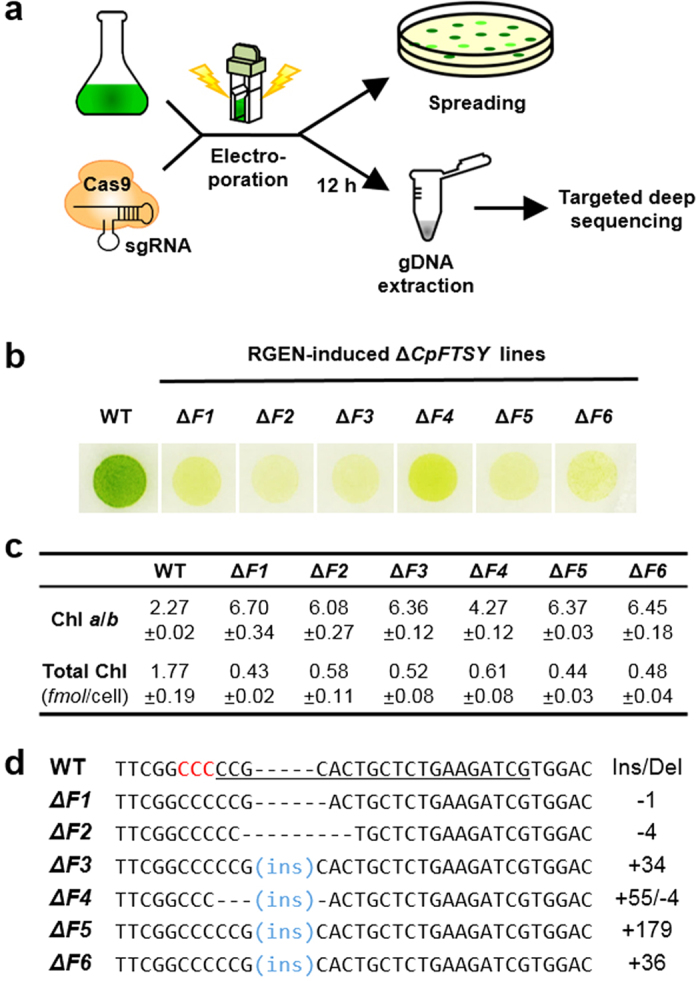
RGEN RNP-mediated target gene disruption in *C. reinhardtii*. (**a**) Experimental process schematic showing the steps applied in this work. (**b**) Single-cell colonies of wild type (WT) and RGEN-induced ΔCpFTSY mutant lines grown on minimal agar medium under low-light (50 μmol photons m^−2^ s^−1^) conditions. (**c**) Chlorophyll (Chl) *a* to Chl *b* ratios and total Chl content of wild type (WT) and the Δ*CpFTSY* mutant lines. Cells were grown photoautotrophically under low light (50 μmol photons m^−2^ s^−1^) conditions. Data are the average and SE from three biological replicates. (**d**) Alignment of wild type (WT) and the mutant line DNA sequences at the *CpFTSY* locus. The 20-bp target sequence is underlined and the PAM sequence is shown in red. The column on the right indicates the number of inserted (+) or deleted (−) bases.

We first generated a specific knockout of the *CpFTSY* gene that confers a smaller, or truncated, chlorophyll (Chl) antenna size of the photosystems. Previously, a null mutation of *CpFTSY* in *C. reinhardtii* caused drastically lower levels of the light-harvesting Chl binding proteins, lower Chl content and a higher Chl *a* to Chl *b* ratio than in wild type^15^. It was suggested that a smaller antenna size might prevent over absorption of sunlight, help greater light penetration of sunlight deeper into a high-density culture, and thus contributing to greater productivity of mass culture under bright sunlight^16^. To this end, we carefully designed four small guide RNAs (sgRNAs) that would cause microhomology-driven frameshift mutations in the target gene using Cas-Designer (www.rgenome.net/cas-designer)^17^,^18^ (Supplementary Fig. 1). We then transfected RGEN-RNPs into *C. reinhardtii* cells (10^7^ cells) by electroporation and streaked them out on a Petri dish (Fig. 1a).

As a result, small insertions and deletions (indels), detected by targeted deep sequencing, were induced at frequencies of up to 0.56% (Supplementary Fig. 2). From RGEN-transfected colonies in each Petri dish (hundreds of colonies per dish), we picked several putative *CpFTSY* knockout cells by visual coloration examination (Supplementary Fig. 3). We confirmed six Δ*CpFTSY* mutants by confirming pale green color (Fig. 1b), by measuring a higher Chl *a* to Chl *b* ratio compared to that in the wild type (Fig. 1c), and by performing Sanger sequencing (Fig. 1d and Supplementary Fig. 4), in each isolated line. For all Δ*CpFTSY* mutants we obtained, total amounts of Chl were three times lower but Chl *a* to Chl *b* ratios were two or three times higher than those of wild type (Fig. 1c). In addition, indel patterns were measured at the expected positions, 3 nt upstream of protospacer–adjacent motif (PAM) sequence (Fig. 1d), indicating the targeted gene mutations by CRISPR RGENs. Notably, we obtained targeted Δ*CpFTSY* mutants more efficiently and directly by this one-step method, compared to the time consuming conventional generation of knock out mutants^19^.

### Targeted *ZEP* gene knockout using RGEN RNPs

**Figure 2:**
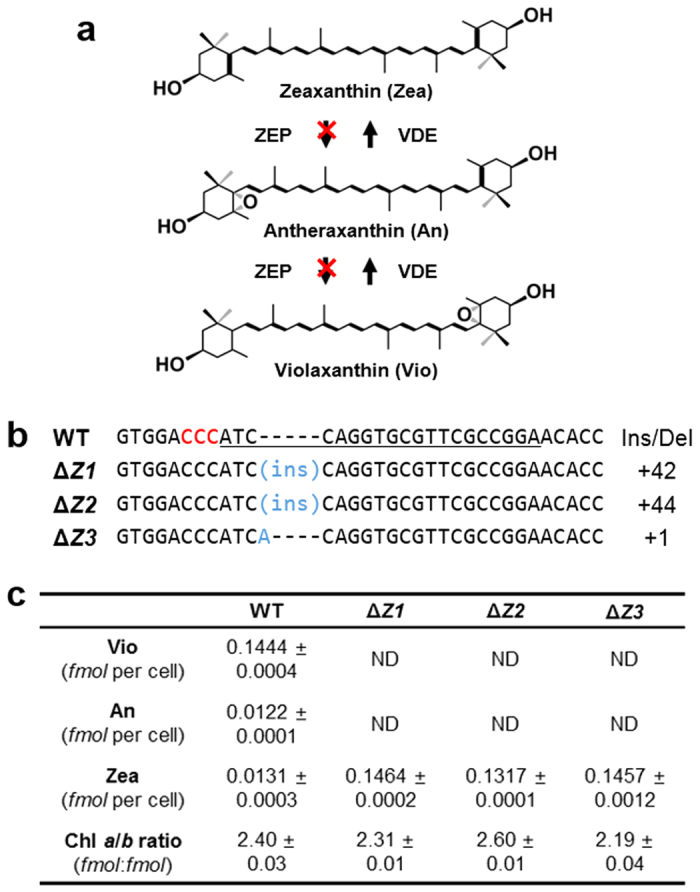
ZEP gene knockout via DNA-free RGEN RNPs delivery. (**a**) Schematic diagram of the reversible xanthophyll cycle in green algae. (**b**) Alignment of wild type (WT) and the mutant line DNA sequences at the *ZEP* locus. The 20-bp target sequence is underlined and the PAM sequence is shown in red. The column on the right indicates the number of inserted (+). (**c**) Quantification of pigment content and Chl *a* to Chl *b* ratios of wild type (WT) and the Δ*ZEP* mutant lines. Cells were grown TAP media under low light (50 μmol photons m^−2^ s^−1^) conditions. Vio, violaxanthin; An, antheraxanthin; Zea, zeaxanthin. Data are the average and SE from three replicates.

We next applied RGEN-RNPs to block zeaxanthin (Zea) epoxidation and, thereby, to accumulate this xanthophyll in *C. reinhardtii*. Zea is a macular pigment of retina, which can prevent the development of chronic diseases such as age-related macular degeneration by filtering hazardous blue light and UV^20^. Blocking the epoxidation step from Zea to violaxanthin (Vio), catalyzed by the zeaxanthin epoxidase (ZEP), can lead to constitutive accumulation of Zea^21^,^22^ (Fig. 2a). Accordingly, we designed five sgRNAs in the *ZEP* locus (Supplementary Fig. 5) as described above and transfected each sgRNA with the purified Cas9 protein into *C. reinhardtii* cells. Targeted deep sequencing showed that indels were induced at a frequency of 0.46% (Supplementary Fig. 6). We picked several putative *ZEP* knockout cells by measuring the Chl fluorescence of the various colonies (Supplementary Fig. 7) and were able to obtain at least three Δ*ZEP* mutants, confirmed by Sanger sequencing (Fig. 2b and Supplementary Fig. 8) and by measuring the amounts of Zea and Vio via HPLC (Supplementary Fig. 9). As expected, indel patterns were shown at the expected positions (Fig. 2b) and Zea were significantly increased more than ten times in Δ*ZEP* mutants compared to the wild type, even under low light growth conditions (Fig. 2c).

### Two-gene of *ZEP* and *CpFTSY* knockout using RGEN RNPs

**Figure 3:**
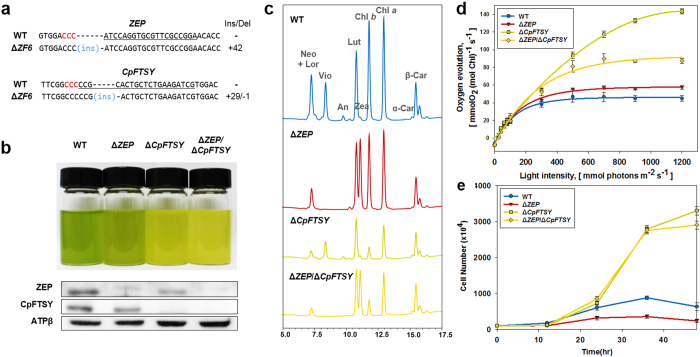
The sequential CpFTSY and ZEP two-gene knockout mutants by transfecting RGEN-RNPs. (**a**) Targeted indel mutations induced by RGEN RNPs at the *ZEP* and *CpFTSY* gene in the Δ*ZEP*/Δ*CpFTSY* double mutant. (**b**) Phenotype of wild type, Δ*ZEP*, Δ*CpFTSY* and Δ*ZEP*/Δ*CpFTSY* mutants of C. reinhardtii. Cells were grown photoautotrophically under high-light (500 μmol photons m^−2^ s^−1^) conditions. Cell densities were 10 × 10^6^ cells/mL. Western-blot analysis of the ZEP and FTSY proteins in the wild type (WT) and the RGEN-induced transgenic lines. Immuno-detection of proteins with the β-subunit of ATP synthase (ATPβ) of *Chlamydomonas* was used as the loading control of the samples. (**c**) HPLC profiles of total pigments from acetone extracts of wild type(blue), Δ*ZEP*(red), Δ*CpFTSY*(green) and Δ*ZEP*/Δ*CpFTSY* double mutant(yellow). Lor, Loroxanthin; Neo, neoxanthin; Vio, violaxanthin; An, antheraxanthin; Lut, lutein; Zea, zeaxanthin; Chl *b*, Chlorophyll *b*; Chl *a*, Chlorophyll *a*; α-Car, α -carotene; β-Car, β-carotene. (**d**) Light-saturation curves of photosynthesis in wild type (blue) and Δ*ZEP* (red), Δ*CpFTSY* (green) and Δ*ZEP*/Δ*CpFTSY* (yellow). Rates of oxygen evolution on a per Chl basis were measured as a function of incident light intensity. (**e**) Growth curve of wild type and the mutants lines cultured in HS medium at 25 °C with air containing 5% CO_2_ under High light (700 μmol photons m^−2^ s^−1^).

We then sought to combine the two genotypes to enhance productivity and to accumulate Zea in mass culture under bright sunlight conditions. Thus, we transfected RGEN-RNPs targeted to *CpFTSY* into one of Δ*ZEP* mutants, Δ*Z1* in Fig. 2b, to obtain two-gene knockout mutants. As a result, indels were measured by targeted deep sequencing at the target site with a frequency of 1.1% (Supplementary Fig. 10). We were ultimately able to obtain fourteen Δ*ZEP*/Δ*CpFTSY* mutant lines by confirming pale green color (Supplementary Fig. 11), by performing Sanger sequencing (Supplementary Fig. 12), and by measuring a higher Chl *a* to Chl *b* ratio compared to that in the wild type (Supplementary Table 1). One of the Δ*ZEP*/Δ*CpFTSY* mutants which have indel patterns at target loci (Fig. 3a and Δ*ZF6* in Supplementary Fig. 12) showed that Zea was accumulated similarly as in the Δ*ZEP* single mutant, while the total amount of Chl was significantly lower that the Δ*ZEP* single mutant and comparable to that in the Δ*CpFTSY* mutant (Fig. 3b). Despite the total absence of ZEP and CpFTSY proteins, confirmed by Western blot analysis, Δ*ZEP*/Δ*CpFTSY* lines could grow in the light, attaining golden rather than green coloration (Fig. 3c), caused by the lower Chl and greater Zea content.

To confirm that the phenotypes in the double mutant cells were not caused by off-target effects, we investigated whether the *CpFTSY* and *ZEP*–targeted RGENs induced off-target mutations. We identified potential off-target sites that differed from on-target sites by 4 nucleotides with no bulge and by 2 nucleotides with one DNA or RNA bulge using Cas-OFFinder^23^ (www.rgenome.net/cas-offinder). No indels were found at these sites above sequencing error rates, in line with previous studies (Supplementary Fig. 13)^24^.

### Photosynthetic productivity of two-gene knockout mutant

We finally compared the photosynthetic productivity of the Δ*ZEP*/Δ*CpFTSY* double mutant with that of the Δ*ZEP* single mutant. The Chl antenna size of the photosystems is well known to impact the photosynthetic rate of mutants and wild type^16^. The quantum yields of photosynthesis of Δ*ZEP*/Δ*CpFTSY*, Δ*ZEP* and wild type were essentially same, indicating that this parameter was not affected by single or double mutation (Fig. 3d). However, the maximum photosynthetic rate (P_max_) on a Chl basis of the Δ*ZEP*/Δ*CpFTSY* mutant was about 54% greater than that of the Δ*ZEP* mutant at saturating irradiance. This difference is attributed to the truncated antenna size of the Δ*ZEP*/Δ*CpFTSY* mutant, indicating a higher productivity per Chl in high light (HL) conditions. Furthermore, growth of the Δ*ZEP*/Δ*CpFTSY* mutant line under HL conditions was dramatically better than that of the wild type and the Δ*ZEP* mutant (Fig. 3e). Therefore, the Δ*ZEP*/Δ*CpFTSY* double mutant showed greater photosynthetic activity and greater light use efficiency than the Δ*ZEP* mutant, suggesting that the double mutant could lead to greater biomass accumulation under HL growth conditions. Photosynthetic activities of the wild type, Δ*ZEP*, Δ*CpFTSY* and Δ*ZEP*/Δ*CpFTSY* mutants were summarized in Table 1.

In conclusion, we achieved DNA-free targeted gene editing in *C. reinhardtii*. This simple RGEN RNP method can be applied to other microalgae without a requirement of a time-consuming cloning step. Moreover, the resulting transformants would be except from GMO regulation, enabling applications of microalgae for pharmaceutical, nutraceutical, food and animal feed and in the medical treatment of specific diseases.

## Methods

### Cell cultivation

*Chlamydomonas reinhardtii* strains CC-4349 cw15 mt- and the mutant strains were cultivated and maintained as described previously^25^. Briefly, Cells were maintained at 25 °C under continuous white light at low light (50 μmol photons m^−2^ s^−1^). Cells were cultivated photoheterotrophically in Tris-acetate phosphate (TAP) medium, or photoautotrophically in high-salt (HS) medium under continuous low and high irradiance (50 and 700 μmol photons m^−2^ s^−1^, respectively). Data in all experiments indicate mean the average and SE from at least three biological replicates.

### RNA preparation

Purified recombinant Cas9 protein was purchased from ToolGen, Inc. Guide RNA was transcribed *in vitro* using the MEGAshortscript T7 kit (Ambion) as previously described^9^. Transcribed RNA was purified by phenol:chloroform extraction, chloroform extraction, and ethanol precipitation. Purified RNA was quantified by spectrometry.

### *Chlamydomonas* transformation

To generate target-specific knockout mutants using RNP complex in *Chlamydomonas*, 50 × 10^4^ cells were transformed with Cas9 protein (200 μg) premixed with *in vitro* transcribed sgRNA (140 μg). Cas9 protein in storage buffer (20 mM HEPES pH 7.5, 150 mM KCl, 1 mM DTT, and 10% glycerol) was mixed with sgRNA dissolved in nuclease-free water and incubated for 10 minutes at room temperature. Cells were transformed with a Biorad Gene Pulser Xcell™ Electroporation Systems, according to the recommended protocol from the GeneArt Chlamydomonas Engineering kit. After transformation, cells were incubated 12 hours and harvested for genomic DNA extraction, or immediately diluted the number of cells 2000 and plated on TAP medium containing 1.5% agar to obtain single colonies for further investigation (Supplementary Fig. 14).

### Targeted deep sequencing

Genomic DNA segments that encompass the nuclease target sites were amplified using Phusion polymerase (New England Biolabs). Equal amounts of the PCR amplicons were subjected to paired-end read sequencing using Illumina MiSeq. Rare sequence reads that occur only once were excluded to remove errors associated with sequencing reaction and amplification. Indels located around the RGEN cleavage site (3 bp upstream of the PAM) were considered to be mutations induced by RGENs. The deep sequencing data are available at the NCBI Sequence Read Archive (www.ncbi.nlm.nih.gov/sra) under accession number PRJNA327012.

### Examination of potential off-target sites

To examine whether there were nuclease-induced indels at hundreds of thousands of potential off-target sites in each genome sequence, we used Cas-OFFinder^23^ to list potential off-target sites that differed from on-target sites by up to 4 nucleotides or that differed by up to 2 nucleotides with a DNA or a RNA bulge in length. As a result, 17 potential off-target sites were obtained and we carried out targeted deep sequencing in independent clones.

### Genotypic characterization

The genomic DNA was extracted from the cells harvested after transformation, or the individual colonized mutants selected from TAP agar plate, for targeted deep sequencing and Sanger sequencing, respectively. To isolate genomic DNA, cells were harvested, resuspended in a microprep buffer containing 2.5× extract buffer (0.35 M Sorbitol, 0.1 M Tris/HCl pH 7.5, and 5 mM EDTA), 2.5× nuclei lysis buffer (0.2 M Tris/HCl pH 7.5, 0.05 M EDTA, 2 M NaCl, and 2% (w/v) CTAB), and 1 × 5% N-Lauroylsarcosine, and incubated for 2 hr at 65 °C. The genomic DNA was extracted with Chloroform:Isoamylalcohol 24:1 and precipitated with Isopropanol. For Sanger sequencing, the target regions were PCR amplified with specific primers (Supplementary Table 2). The PCR products were verified by agarose gel electrophoresis, eluted from the gel and sequenced using the Sanger method (Macrogen, South Korea).

### Mutant Selection

The targeted knockout mutants were selected upon the basis of coloration of the cells and measurement of chlorophyll (Chl) fluorescence by Walz image-PAM system M-series Chl Fluorescence System equipped with a CCD camera as described^21^. Initially screened transformants were measured pigments to verify their changed pigment compositions.

### Pigments and photosynthetic activity determination

Cells for pigments determination were grown under the low light conditions, 50 μmol photons m^−2^ s^−1^. The Chl content of the cells was spectrophotometrically determined in 80% (v/v) acetone extracts as described^26^,^27^. HPLC analysis was conducted with a Shimadzu Prominence HPLC model LC-20AD equipped with a Waters Spherisorb 5.0 μm ODS1 4.6 × 250 mm cartridge column. The pigment was extracted in 90% (v/v) acetone and the supernatant of sample was subjected to HPLC analysis. The pigments were separated using a solvent mixture of 0.1 M Tris-HCl pH 8.0, acetonitrile, methanol, and ethylacetate. During the run, the solvent concentrations were 14% 0.1 M Tris-HCl, 84% acetonitrile, and 2% methanol from 0 to 15 minutes. From 15 to 19 minutes, the solvent mixture was consisted of 68% methanol and 32% acetonitrile. A post-run was performed for 6 minutes with the initial solvent mixture. The flow rate was constant at 1.2 mL per minute. Pigments were detected at 445 nm and 670 nm. Concentration of the individual pigment was determined from the HPLC profiles calibrated with standard pigments of Chl and carotenoids (14C Centralen; DHI, Hørsholm, Denmark). The photosynthetic activity was measured at 25 °C with a Hansatech Clark-type oxygen electrode illuminated with a halogen lamp. An aliquot of 1 mL cell suspension containing 2 μM Chl was transferred to the oxygen electrode chamber. To ensure that oxygen evolution was not limited by the carbon supply available to the cells, 50 μL of 0.5 M NaHCO_3_, pH 7.4 was added to the suspension before the measurements. The oxygen evolution was measured at increasing light intensities, 0 to 1,200 μmol photons m^−2^ s^−1^, and each step was recorded for 2 minutes.

### SDS-PAGE and Western-Blot Analysis

For protein analysis, cells grown in TAP under 70 μmol photons m^−2^ s^−1^ were harvested, resuspended in lysis buffer (20 mM HEPES-KOH pH 7.5, 5 mM MgCl_2_, 5 mM β-mercaptoethanol and 1 mM PMSF) and disrupted by sonication. Total protein samples loaded and separated on SDS-PAGE gel as described26. The SDS-PAGE gels were stained with 0.1% (w/v) Coomassie Brilliant Blue R for visualization, or blotted onto an ATTO P PVDF membrane via a semi dry transfer system. Membranes were probed with specific polyclonal antibodies raised against the Zeaxanthin epoxidase (ZEP), cpFTSY and β subunit of ATP synthase (ATPβ) from *C. reinhardtii*. Signals were visualized using the Abfrontier west save up ECL Reagent and exposed to an X-ray film for signal detection.

### Growth analysis

*Chlamydomonas* cells were photoautotrophically grown in 200 mL HS media in the bubble column photobioreactor (40 mm in diameter and 500 mm in height) illuminated with continuous high light (700 μmol photons m^−2^ s^−1^) at 25 °C. Each culture was aerated with 5% CO_2_ at a feed velocity of 40 mL/min. The initial cell concentration for each culture was 100 × 10^4^ cells/mL.

## Additional Information

**How to cite this article**: Baek, K. *et al*. DNA-free two-gene knockout in *Chlamydomonas reinhardtii* via CRISPR-Cas9 ribonucleoproteins. *Sci. Rep.* **6**, 30620; doi: 10.1038/srep30620 (2016).

## Supplementary Material

Supplementary Information

## Figures and Tables

**Table 1:** Quantification of pigment content and photosynthetic activities of the wild type, Δ*ZEP*, Δ*CpFTSY* and Δ*ZEP*/Δ*CpFTSY* mutants.

	WT	Δ*ZEP*	Δ*CpFTSY*	Δ*ZEP*/Δ*CpFTSY*
Vio* (fmol per cell)	0.1043 ± 0.0010	ND	0.0551 ± 0.0003	ND
An* (fmol per cell)	0.0085 ± 0.0002	ND	0.0033 ± 0.0002	ND
Zea* (fmol per cell)	0.0062 ± 0.0005	0.1376 ± 0.0007	0.0056 ± 0.0002	0.0827 ± 0.0009
Total Chl* (fmol per cell)	1.66 ± 0.01	1.41 ± 0.03	0.51 ± 0.01	0.60 ± 0.01
Chl a/b ratio* (fmol:fmol)	2.24 ± 0.02	2.27 ± 0.02	6.22 ± 0.15	6.14 ± 0.09
**Quantum yield** (relative units)	100 ± 3.63	109.05 ± 14.36	117.09 ± 8.10	109.85 ± 8.59
**P**_**max**_ (mmol oxygen (mol Chl)^−1^ s^−1^)	48.93 ± 0.76	61.4 ± 2.12	143.28 ± 3.24	94.77 ± 2.21

^*^Pigments were measured under low-light (50 μmol photons m^−2^ s^−1^) conditions. Vio, violaxanthin; An, antheraxanthin; Zea, zeaxanthin.

Data are the average and SE from three replicates.

## References

[b1] Wijffels R. H. & Barbosa M. J. An outlook on microalgal biofuels. Science 329, 796–799 (2010). 20705853 10.1126/science.1189003

[b2] Koller M., Muhr A. & Braunegg G. Microalgae as versatile cellular factories for valued products. Algal Res. 6, 52–63 (2014).

[b3] Sizova I., Greiner A., Awasthi M., Kateriya S. & Hegemann P. Nuclear gene targeting in Chlamydomonas using engineered zinc‐finger nucleases. Plant J. 73, 873–882 (2013). 23137232 10.1111/tpj.12066

[b4] Daboussi F. . Genome engineering empowers the diatom *Phaeodactylum tricornutum* for biotechnology. Nat Commun. 5, (2014). 10.1038/ncomms4831 24871200

[b5] Gao H. . TALE activation of endogenous genes in *Chlamydomonas reinhardtii*. Algal Res. 5, 52–60 (2014).

[b6] Jiang W., Brueggeman A. J., Horken K. M., Plucinak T. M. & Weeks D. P. Successful transient expression of Cas9 and single guide RNA genes in *Chlamydomonas reinhardtii*. Eukaryot Cell 13, 1465–1469 (2014). 25239977 10.1128/EC.00213-14 PMC4248704

[b7] Kim H. & Kim J.-S. A guide to genome engineering with programmable nucleases. Nat Rev Genet. 15, 321–334 (2014). 24690881 10.1038/nrg3686

[b8] Cho S. W., Lee J., Carroll D., Kim J.-S. & Lee J. Heritable gene knockout in *Caenorhabditis elegans* by direct injection of Cas9–sgRNA ribonucleoproteins. Genetics 195, 1177–1180 (2013). 23979576 10.1534/genetics.113.155853 PMC3813847

[b9] Kim S., Kim D., Cho S. W., Kim J. & Kim J.-S. Highly efficient RNA-guided genome editing in human cells via delivery of purified Cas9 ribonucleoproteins. Genome Res. 24, 1012–1019 (2014). 24696461 10.1101/gr.171322.113 PMC4032847

[b10] Zuris J. A. . Cationic lipid-mediated delivery of proteins enables efficient protein-based genome editing *in vitro* and *in vivo*. Nat Biotechnol 33, 73–80 (2015). 25357182 10.1038/nbt.3081 PMC4289409

[b11] Woo J. W. . DNA-free genome editing in plants with preassembled CRISPR-Cas9 ribonucleoproteins. Nat Biotechnol. 33, 1162–1164 (2015). 26479191 10.1038/nbt.3389

[b12] Subburaj S. . Site-directed mutagenesis in Petunia × hybrida protoplast system using direct delivery of purified recombinant Cas9 ribonucleoproteins. Plant Cell Rep. 35, 1535–1544 (2016). 26825596 10.1007/s00299-016-1937-7

[b13] Kanchiswamy C. N., Malnoy M., Velasco R., Kim J.-S. & Viola R. Non-GMO genetically edited crop plants. Trends Biotechnol. 33, 489–491 (2015). 25978870 10.1016/j.tibtech.2015.04.002

[b14] Merchant S. S. . The *Chlamydomonas* genome reveals the evolution of key animal and plant functions. Science 318, 245–250 (2007). 17932292 10.1126/science.1143609 PMC2875087

[b15] Kirst H., García-Cerdán J. G., Zurbriggen A. & Melis A. Assembly of the light-harvesting chlorophyll antenna in the green alga *Chlamydomonas reinhardtii* requires expression of the TLA2-CpFTSY gene. Plant Physiol 158, 930–945 (2012). 22114096 10.1104/pp.111.189910 PMC3271779

[b16] Melis A. Solar energy conversion efficiencies in photosynthesis: minimizing the chlorophyll antennae to maximize efficiency. Plant Sci. 177, 272–280 (2009).

[b17] Park J., Bae S. & Kim J.-S. Cas-Designer: a web-based tool for choice of CRISPR-Cas9 target sites. Bioinformatics 31, 4014–4016 (2015). 26358729 10.1093/bioinformatics/btv537

[b18] Bae S., Kweon J., Kim H. S. & Kim J.-S. Microhomology-based choice of Cas9 nuclease target sites. Nat Methods 11, 705–706 (2014). 24972169 10.1038/nmeth.3015

[b19] Kindle K. L. High-frequency nuclear transformation of *Chlamydomonas reinhardtii*. Proc Natl Acad Sci USA 87, 1228–1232 (1990). 2105499 10.1073/pnas.87.3.1228 PMC53444

[b20] Bernstein P. S. . Lutein, zeaxanthin, and meso-zeaxanthin: The basic and clinical science underlying carotenoid-based nutritional interventions against ocular disease. Prog Retin Eye Res. 50, 34–66 (2016). 26541886 10.1016/j.preteyeres.2015.10.003 PMC4698241

[b21] Niyogi K. K., Bjorkman O. & Grossman A. R. *Chlamydomonas* xanthophyll cycle mutants identified by video imaging of chlorophyll fluorescence quenching. Plant Cell 9, 1369–1380 (1997). 12237386 10.1105/tpc.9.8.1369 PMC157004

[b22] Jin E., Feth B. & Melis A. A mutant of the green alga *Dunaliella salina* constitutively accumulates zeaxanthin under all growth conditions. Biotechnol Bioeng 81, 115–124 (2003). 12432587 10.1002/bit.10459

[b23] Bae S., Park J. & Kim J.-S. Cas-OFFinder: a fast and versatile algorithm that searches for potential off-target sites of Cas9 RNA-guided endonucleases. Bioinformatics 30, 1473–1475 (2014). 24463181 10.1093/bioinformatics/btu048 PMC4016707

[b24] Kim D. . Digenome-seq: genome-wide profiling of CRISPR-Cas9 off-target effects in human cells. Nat Methods 12, 237–243 (2015). 25664545 10.1038/nmeth.3284

[b25] Baek K., Lee Y., Nam O., Park S., Sim S. J. & Jin E. Introducing Dunaliella LIP promoter containing light-inducible motifs improves transgenic expression in *Chlamydomonas reinhardtii*. Biotechnology Journal 11, 384–392 (2016). 26773277 10.1002/biot.201500269

[b26] Arnon D. I. Copper enzymes in isolated chloroplasts. Polyphenoloxidase in *Beta vulgaris*. Plant Physiol 24, 1 (1949). 16654194 10.1104/pp.24.1.1 PMC437905

[b27] Melis A., Spangfort M. & Andersson B. Light‐absorption and electron‐transport balance between photosystem II and photosystem I in spinach chloroplasts. Photochem Photobiol 45, 129–136 (1987).

