# Supplementary material for: DNA-free two-gene knockout in Chlamydomonas reinhardtii via CRISPR-Cas9 ribonucleoproteins
Source: Sci Rep. 2016 Jul 28;6:30620. doi: 10.1038/srep30620 (PMC4964356; doi:10.1038/srep30620)
Supplement: Supplementary Information [file srep30620-s1.doc]

**Supplementary Information**

DNA-free two-gene knockout in *Chlamydomonas reinhardtii* *via* CRISPR-Cas9 ribonucleoproteins

Kwangryul Baek^1,7^, Duk Hyoung Kim^2,7^, Jooyeon Jeong^1^, Sang Jun Sim^3^, Anastasios Melis^4^, Jin-Soo Kim^5,6^,*, EonSeon Jin^1^,*, Sangsu Bae^2^,*

^1^Department of Life Science, Hanyang University, Seoul, South Korea,

^2^Department of Chemistry, Hanyang University, Seoul, South Korea,

^3^Department of Chemical and Biological Engineering, Korea University, Seoul, South Korea,

^4^Department of Plant and Microbial Biology, University of California, Berkeley, CA 94720-3102, USA,

^5^Center for Genome Engineering, Institute for Basic Science, Seoul, South Korea

^6^Department of Chemistry, Seoul National University, Seoul, South Korea

^7^These authors contributed equally to this work.

*Correspondence should be addressed to J.-S.K. ([jskim01@snu.ac.kr](mailto:jskim01@snu.ac.kr)), E.S.J. ([esjin@hanyang.ac.kr](mailto:esjin@hanyang.ac.kr)), S.B ([sangsubae@hanyang.ac.kr](mailto:sangsubae@hanyang.ac.kr))

**Table of Contents**

**Supplementary Figures**

Supplementary Figure 1. Description of four sgRNAs for targeting *CpFTSY* gene.

Supplementary Figure 2. RGEN-RNPs mediated mutagenesis of *CpFTSY* gene.

Supplementary Figure 3. Visual coloration examination for hundreds of colonies to investigate *CpFTSY* gene knockout.

Supplementary Figure 4. Chromatograms of six RGEN-induced Δ*CpFTSY* mutant lines.

Supplementary Figure 5. Description of five sgRNAs for targeting *ZEP* gene.

Supplementary Figure 6. RGEN-RNPs mediated mutagenesis of *ZEP* gene.

Supplementary Figure 7. Measurement of the chlorophyll (Chl) fluorescence for hundreds of colonies to investigate *ZEP* gene knockout.

Supplementary Figure 8. Chromatograms of three RGEN-induced Δ*ZEP* mutant lines.

Supplementary Figure 9. HPLC profiles of total pigments from acetone extracts in wild type (blue) and Δ*Z1* (red), Δ*Z2* (magenta) and Δ*Z3* (purple).

Supplementary Figure 10. RGEN-RNPs mediated sequential mutagenesis of *CpFTSY* gene in Δ*ZEP* mutant line.

Supplementary Figure 11. Visual coloration examination for hundreds of colonies to investigate *CpFTSY* gene knockout in Δ*ZEP* mutant line.

Supplementary Figure 12. Chromatograms of fourteen RGEN-induced Δ*ZEP*/Δ*CpFTSY* mutant lines.

Supplementary Figure 13. Analysis of off-target effects in one Δ*ZEP*/Δ*CpFTSY* mutant line.

Supplementary Figure 14. Mutation frequencies at different incubation time and various concentration conditions of Cas9 protein and sgRNA.

**Supplementary Tables**

Supplementary Table 1. Quantification of pigment content, total Chl and Chl *a* to Chl *b* ratios of WT, Δ*ZEP* mutant and the Δ*ZEP*/Δ*CpFTSY* mutant lines.

Supplementary Table 2. Primers used in this study.

**Supplementary Figures**

**Supplementary Figure 1. Description of four sgRNAs for targeting *CpFTSY* gene.** We carefully designed four sgRNAs within half of coding sequence region of *CpFTSY* gene using Cas-Designer ([www.rgenome.net/cas-designer/](http://www.rgenome.net/cas-designer/)), that differed from any other target sites in whole genome by 3 nucleotides (nt) and had higher out-of-frame scores than 66. The ‘CDS (coding sequence) position’ means the relative position of the cleavage point in RNA transcript. The ‘out-of-frame score’ indicates the probability of frameshift-inducing deletions occurring when broken double-stranded DNA is repaired by the microhomology-mediated end joining (MMEJ) pathway. The ‘# of off-target sites’ means the number of mismatched sequences throughout the whole genome.


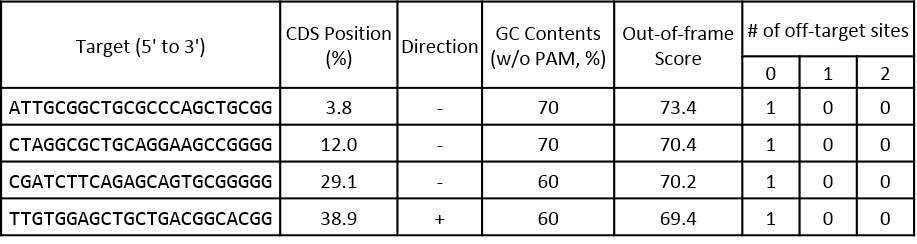


**Supplementary Figure 2. RGEN-RNPs mediated mutagenesis of *CpFTSY*.** (a) Mutation (insertion and deletion; indel) frequency of wild type and RGEN-transfected cells for each sgRNA were measured by targeted deep sequencing. For the sgRNA with asterisk mark (*) was excluded from targeted deep sequencing analysis due to non-specific products during PCR amplification. (b) Second trial for most efficient sgRNA in (a). Indel frequency was measured up to about 0.56%. (c) Represent mutant DNA sequences obtained from (b). Various indel patterns were showed at expected position, 3nt upstream of PAM sequence. The 20-bp target sequence is underlined and the PAM sequence is shown in red.


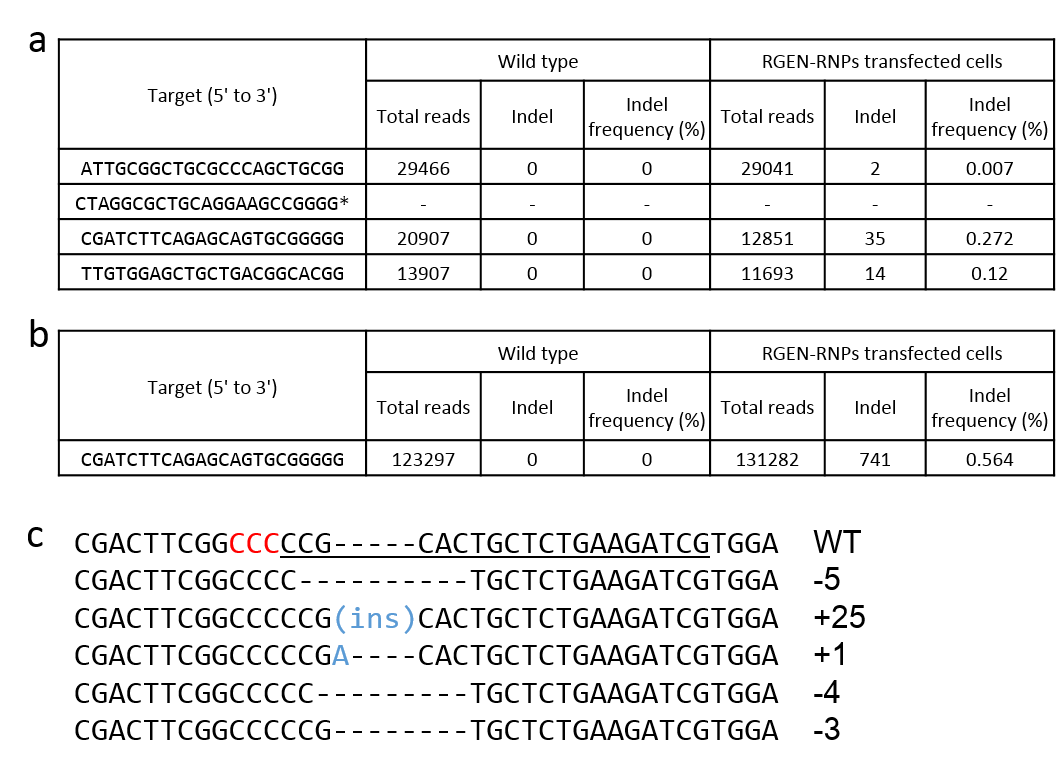


**Supplementary Figure 3. Visual coloration examination for hundreds of colonies to investigate *CpFTSY* gene knockout.** After *CpFTSY* specific knockout mutants were generated using DNA-free RGEN RNPs, we picked several putative *CpFTSY* knockout cell lines which had pale green colors. Red circles indicate the putative *CpFTSY* knockout mutants grown on TAP agar medium under low-light (50 μmol photons m^-2^s^-1^) conditions.


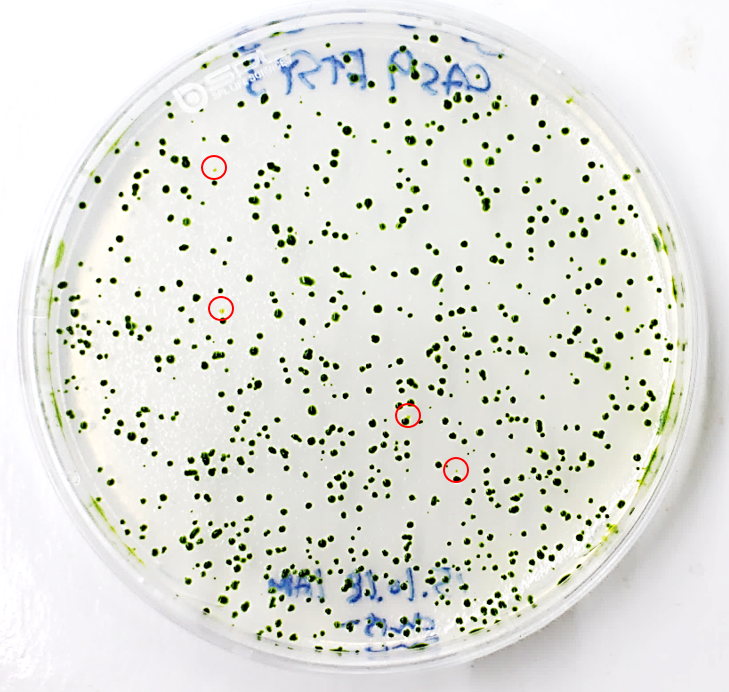


**Supplementary Figure 4. Chromatograms of six RGEN-induced Δ*CpFTSY* mutant lines.** *CpFTSY* gene knockout of six mutant lines shown in Figure 1b were confirmed by performing Sanger sequencing. Various indel patterns were observed at target sites.


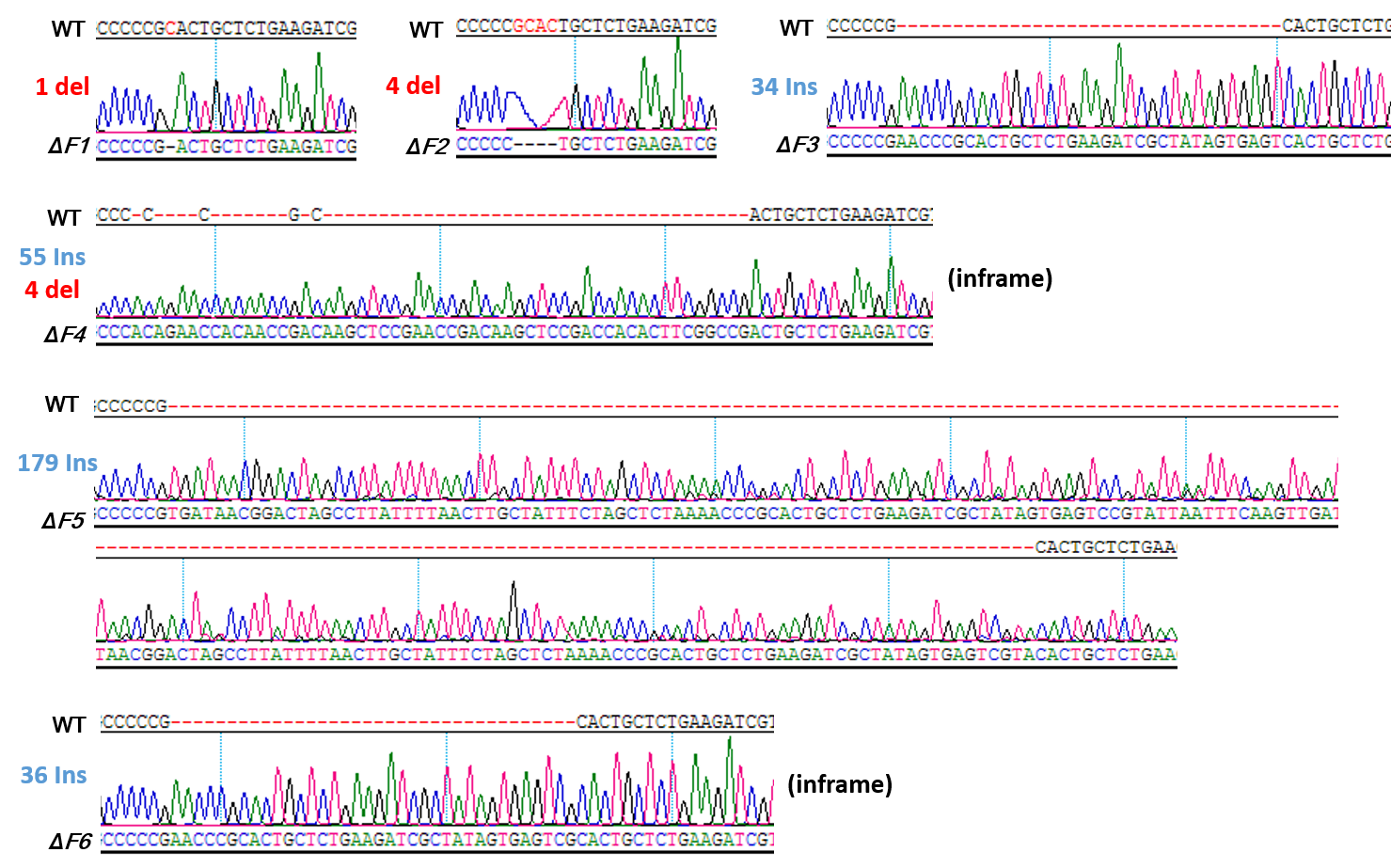


**Supplementary Figure 5. Description of five sgRNAs for targeting *ZEP* gene.** We carefully designed five sgRNAs within half of coding sequence region of *ZEP* gene using Cas-Designer ([www.rgenome.net/cas-designer/](http://www.rgenome.net/cas-designer/)), that differed from any other target sites in whole genome by 3 nucleotides (nt) and had higher out-of-frame scores than 66. The ‘CDS (coding sequence) position’ means the relative position of the cleavage point in RNA transcript. The ‘out-of-frame score’ indicates the probability of frameshift-inducing deletions occurring when broken double-stranded DNA is repaired by the microhomology-mediated end joining (MMEJ) pathway. The ‘# of off-target sites’ means the number of mismatched sequences throughout the whole genome.

**
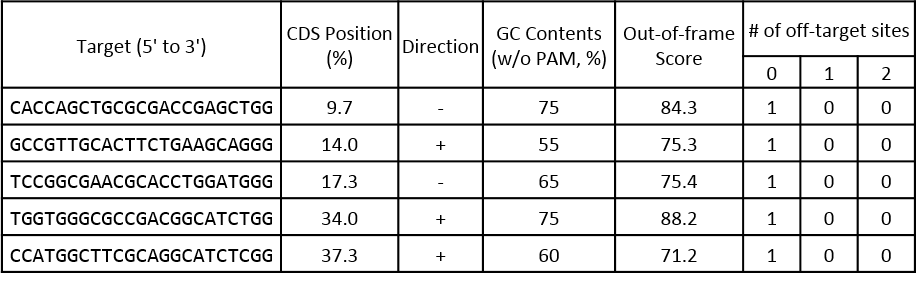
**

**Supplementary Figure 6. RGEN-RNPs mediated mutagenesis of *ZEP*.** (a) Mutation (insertion and deletion; indel) frequency of wild type and RGEN-transfected cells for each sgRNA were measured by targeted deep sequencing. Indel frequency was measured up to about 0.46%. (b) Represent mutant DNA sequences obtained from third sgRNA in (a) i.e. TCCGGCGAACGCACCTGGATGGG. Various indel patterns were shown at expected position, 3nt upstream of PAM sequence. The 20-bp target sequence is underlined and the PAM sequence is shown in red.


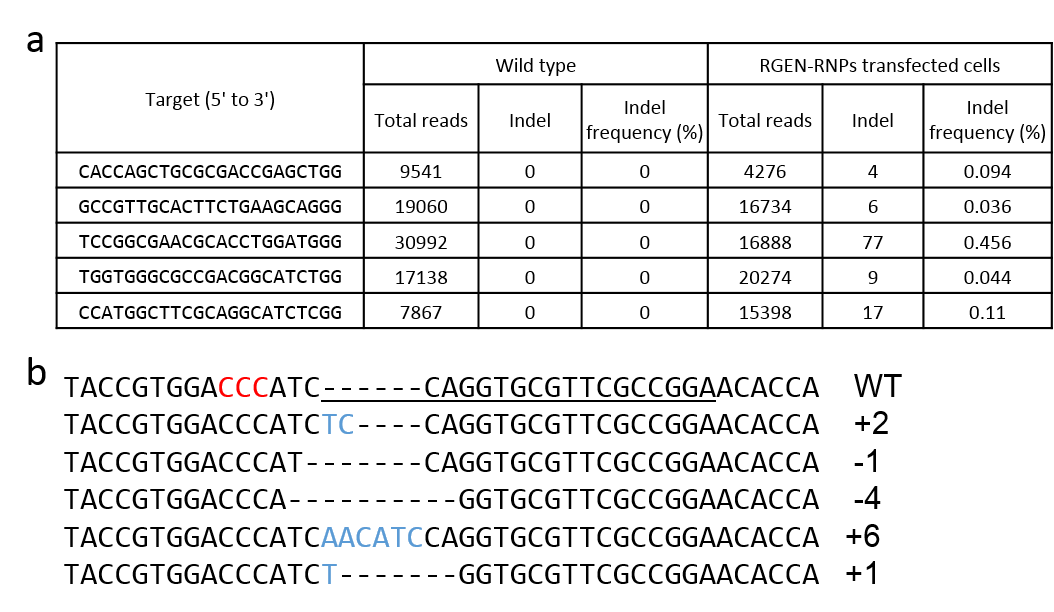


**Supplementary Figure 7. Measurement of the chlorophyll (Chl) fluorescence for hundreds of colonies to investigate *ZEP* gene knockout.** (a) After *ZEP* specific knockout mutants were generated using DNA-free RGEN RNPs, we measured Chl fluorescence for all cells in Petri dish and picked several putative *ZEP* knockout cell lines. Red circle indicates a putative *ZEP* knockout mutant grown on TAP agar medium under low-light (50 μmol photons m^-2^s^-1^) conditions. NPQ/4 images were measured as described in methods. (b) Single cell colonies of wild type (WT) and Δ*ZEP* mutant lines grown on minimal agar medium under low-light (50 μmol photons m^-2^s^-1^) conditions. They showed indistinguishable color appearances.


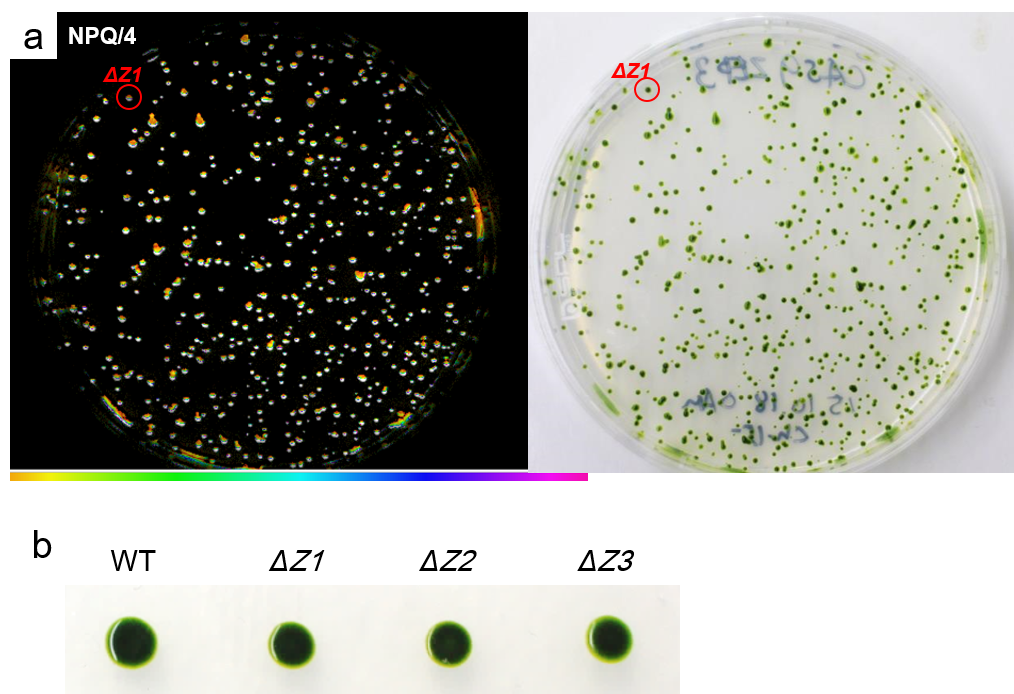


**Supplementary Figure 8. Chromatograms of three RGEN-induced Δ*ZEP* mutant lines.** *ZEP* gene knockout of three mutant lines shown in Supplementary Figure 7 were confirmed by performing Sanger sequencing. Various insertion patterns were observed at target sites.


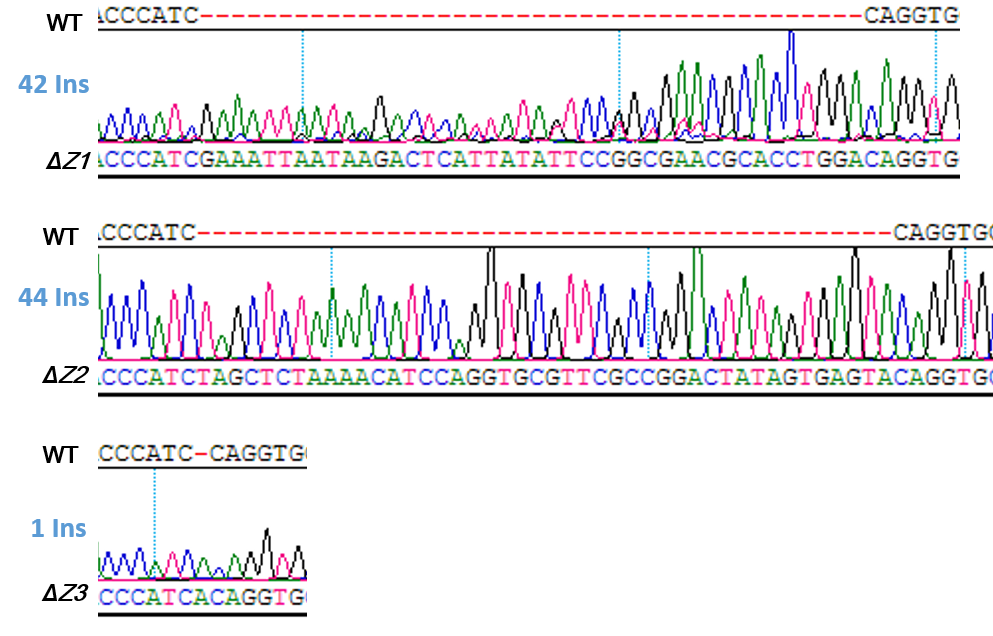


**Supplementary Figure 9. HPLC profiles of total pigments from acetone extracts in wild type (blue) and Δ*Z1* (red), Δ*Z2* (magenta) and Δ*Z3* (purple).** In contrast with wild type, Zeaxanthin was significantly increased in all Δ*ZEP* mutants, even under low light growth conditions. Lor, Loroxanthin; Neo, neoxanthin; Vio, violaxanthin; An, antheraxanthin; Lut, lutein; Zea, zeaxanthin; Chl *b*, Chlorophyll *b*; Chl *a*, Chlorophyll *a*; α-Car, α -carotene; β-Car, β-carotene.

**
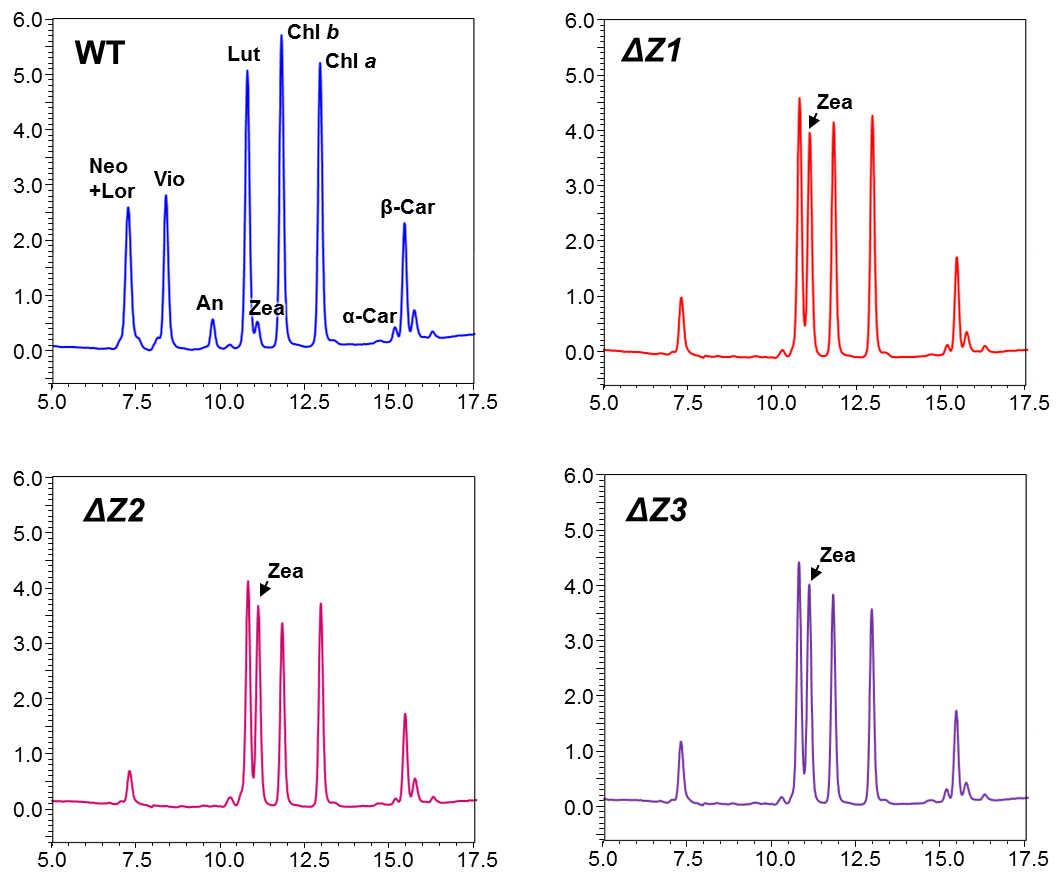
**

**Supplementary Figure 10. RGEN-RNPs mediated sequential mutagenesis of *CpFTSY* gene in Δ*ZEP* mutant line.** (a) Mutation (insertion and deletion; indel) frequency of wild type and RGEN-transfected cells for sgRNA were measured by targeted deep sequencing. Indel frequency was measured up to about 1.11%. (b) Various indel patterns were shown at expected position, 3nt upstream of PAM sequence. The 20-bp target sequence is underlined and the PAM sequence is shown in red.

**
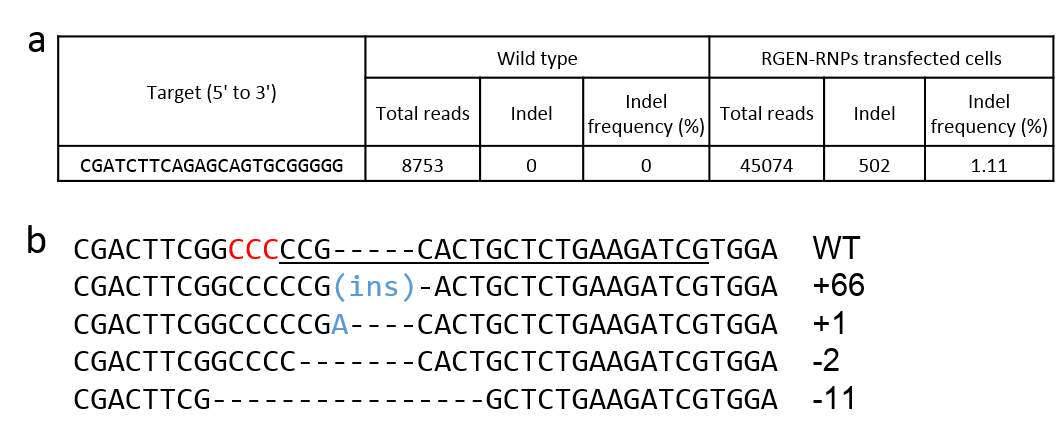
**

**Supplementary Figure 11. Visual coloration examination for hundreds of colonies to investigate *CpFTSY* gene knockout in Δ*ZEP* mutant line.** (a) After *CpFTSY* specific knockout mutants were sequentially generated using DNA-free RGEN RNPs in Δ*ZEP* mutant line, we picked several putative Δ*ZEP*/Δ*CpFTSY* cell lines which had pale green colors. Red circles indicate the putative Δ*ZEP*/Δ*CpFTSY* mutants grown on TAP agar medium under low-light (50 μmol photons m^-2^s^-1^) conditions. (b) Single cell colonies of wild type (WT) and RGEN-induced Δ*ZEP*/Δ*CpFTSY* mutant lines (Δ*ZF1* ~ *14*) grown on minimal agar medium under low-light (50 μmol photons m^-2^s^-1^) conditions.

**
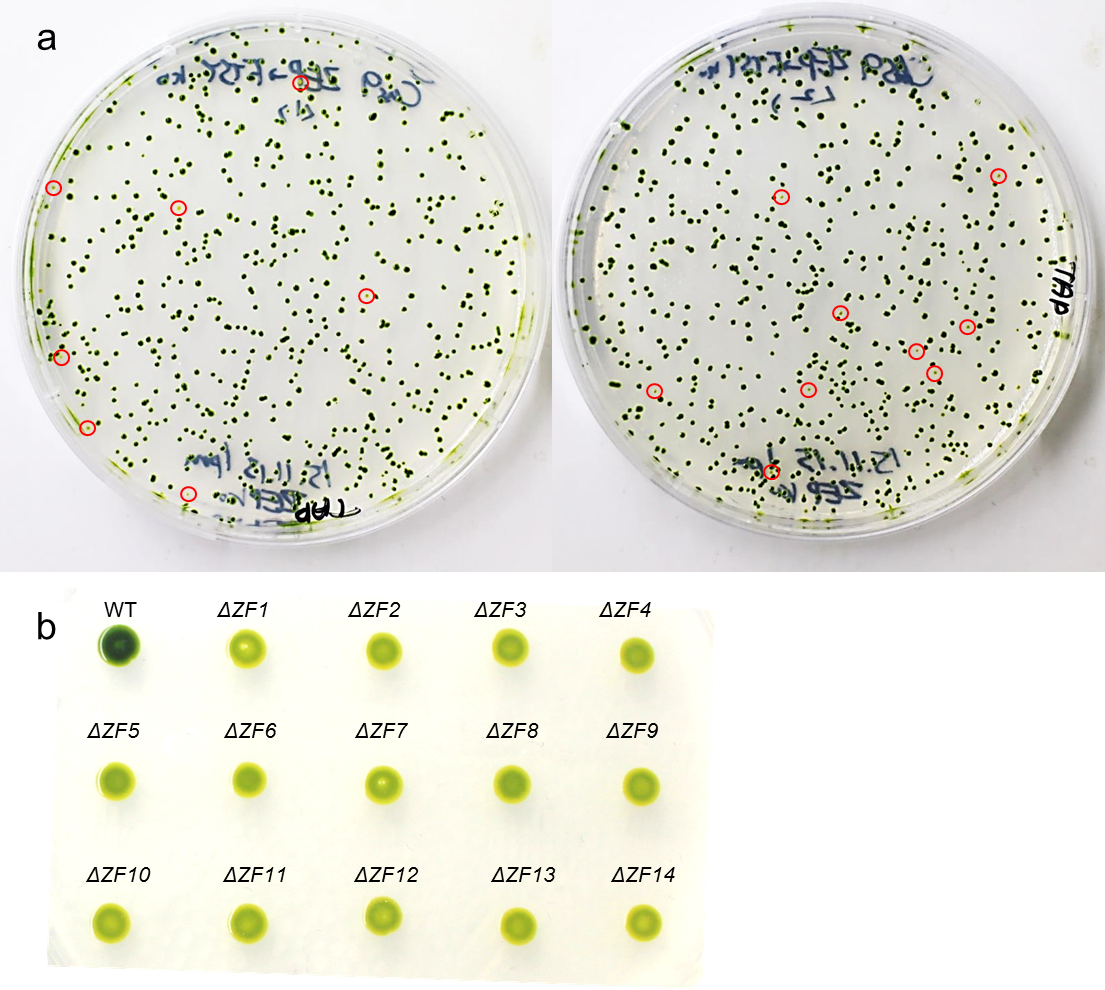
**

**Supplementary Figure 12. Chromatograms of fourteen RGEN-induced Δ*ZEP*/Δ*CpFTSY* mutant lines.** *CpFTSY* gene knockout of fourteen mutant lines shown in Supplementary Figure 11 were confirmed by performing Sanger sequencing. Various insertion patterns were observed at target sites.

**
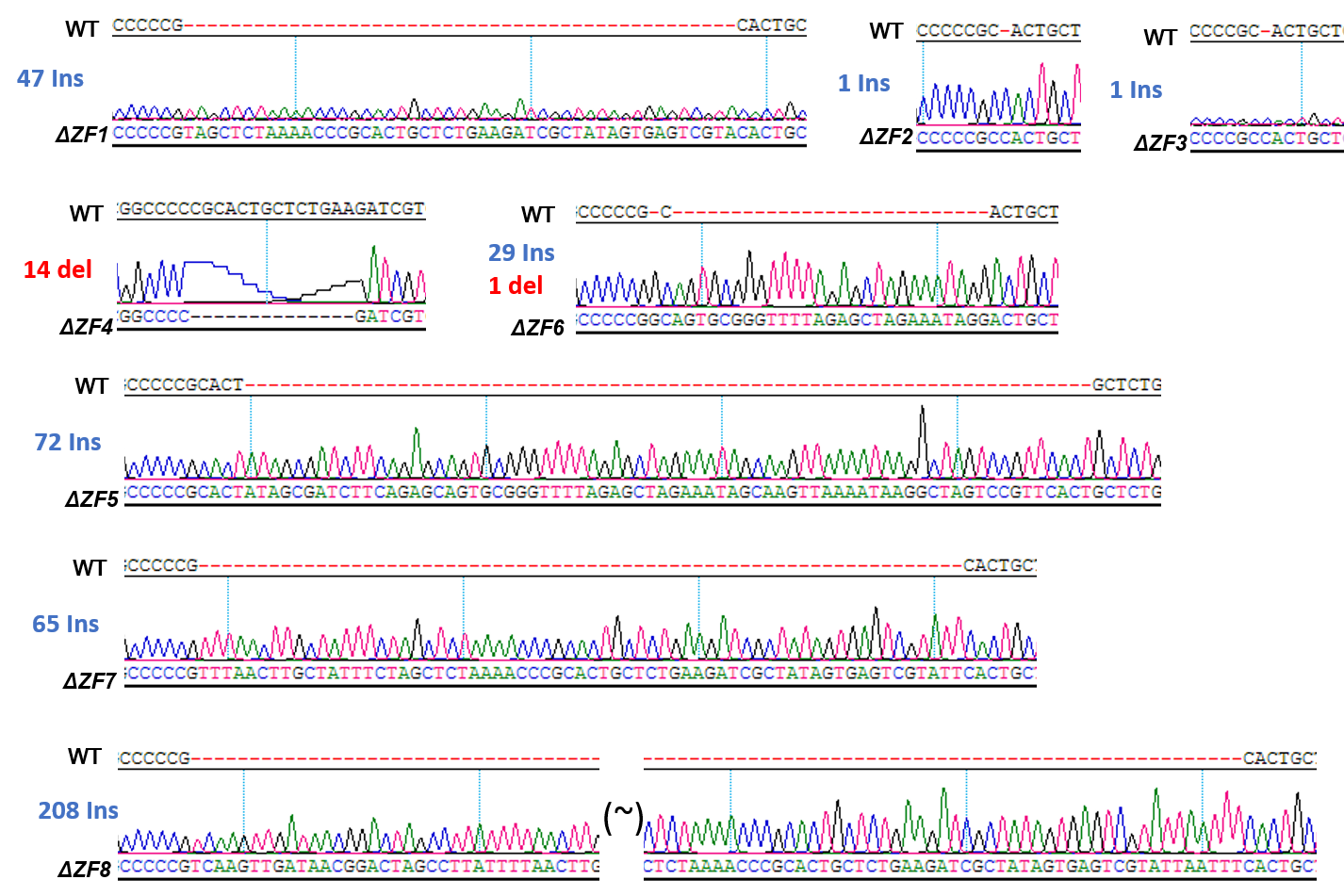

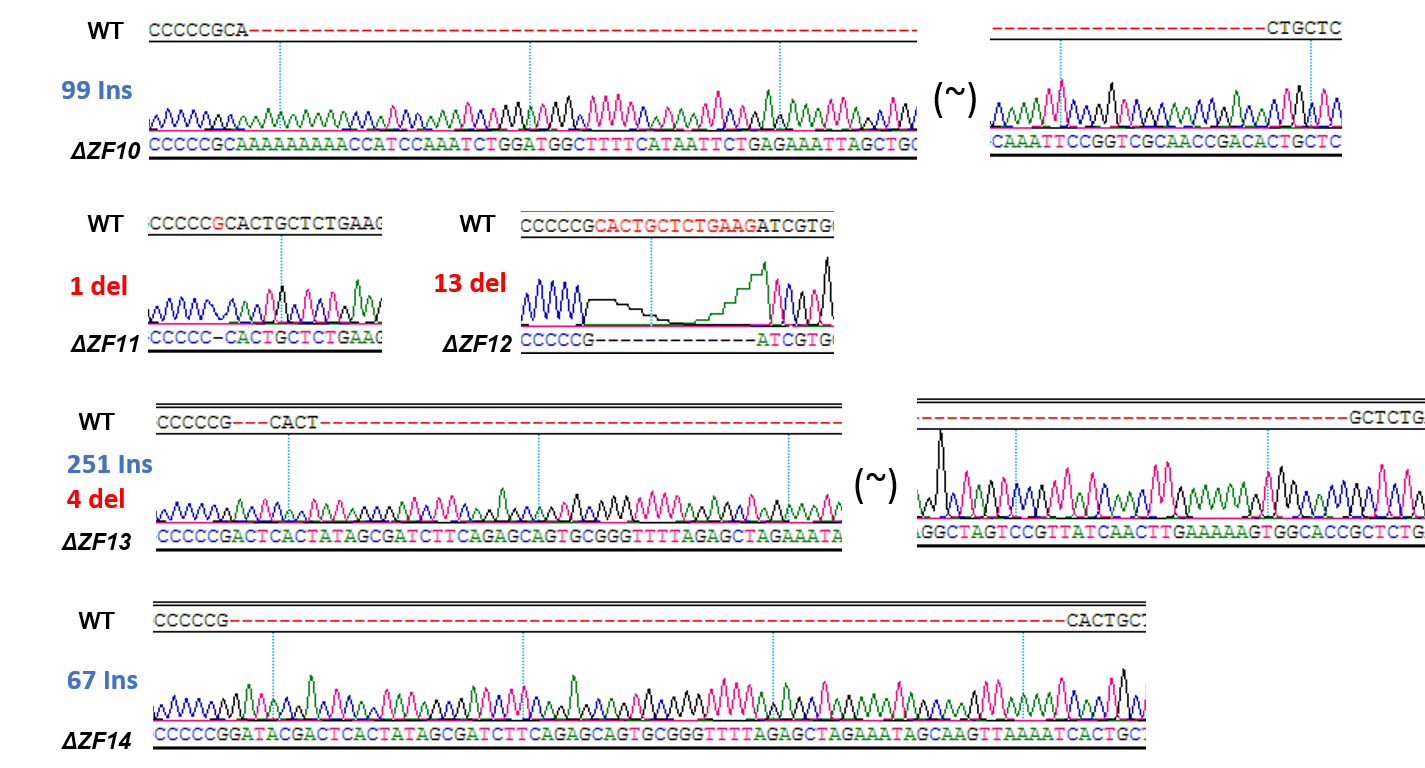
**

**Supplementary Figure 13. Analysis of off-target effects in one Δ*ZEP*/Δ*CpFTSY* mutant line.** Mutation frequencies at on-target and potential off-target sites of the *ZEP* and *CpFTSY* gene-specific sgRNAs were measured by targeted deep sequencing. About ~20,000 paired-end reads per site were obtained to calculate the indel rate.

**
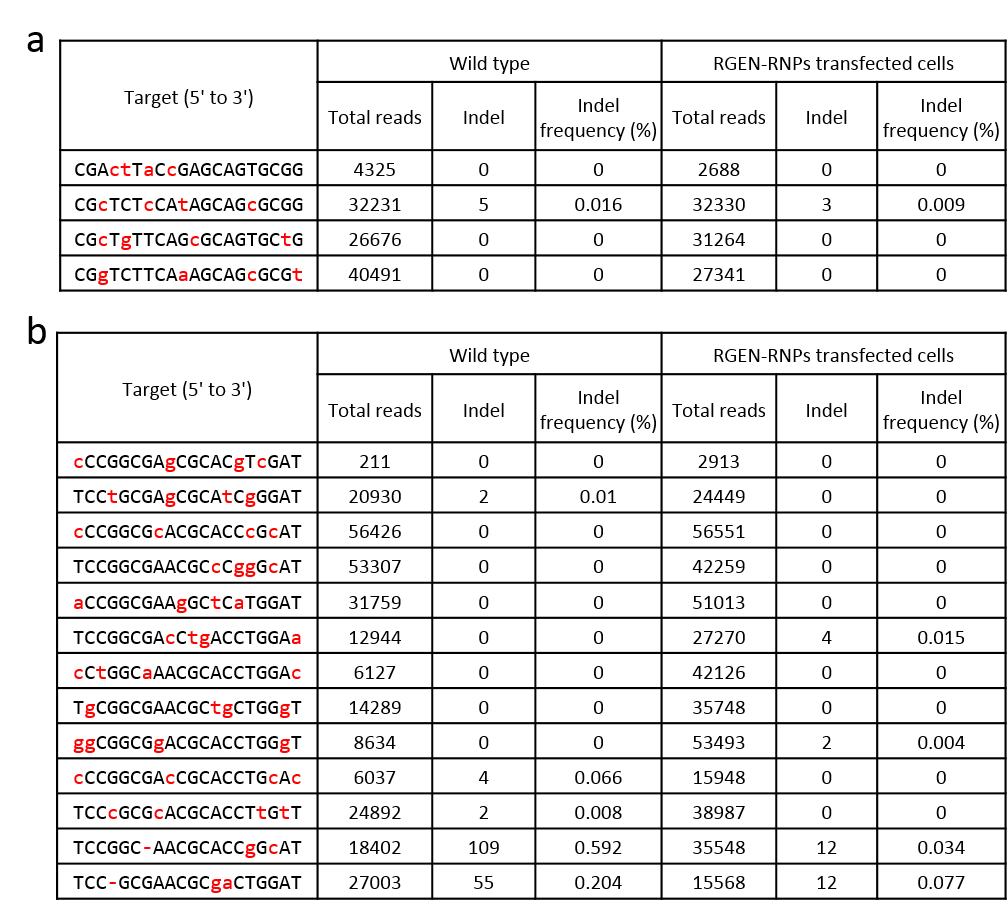
**

**Supplementary Figure 14. Mutation frequencies at different incubation time and various concentration conditions of Cas9 protein and sgRNA.** (a) After RGEN RNPs (200 μg of Cas9 and 140 μg of sgRNA) were transfected into 50ⅹ10^4^ cells of *Chlamydomonas*, they were incubated for different time and harvested. We found no significant difference between the different incubation time of 12h and 24h. (b) Mutation (insertion and deletion; indel) frequencies with various concentration conditions of Cas9 protein and sgRNA were measured by targeted deep sequencing after incubation for 12h.

* Target DNA sequence of sgRNA: 5’- CGATCTTCAGAGCAGTGCGG-3’

**
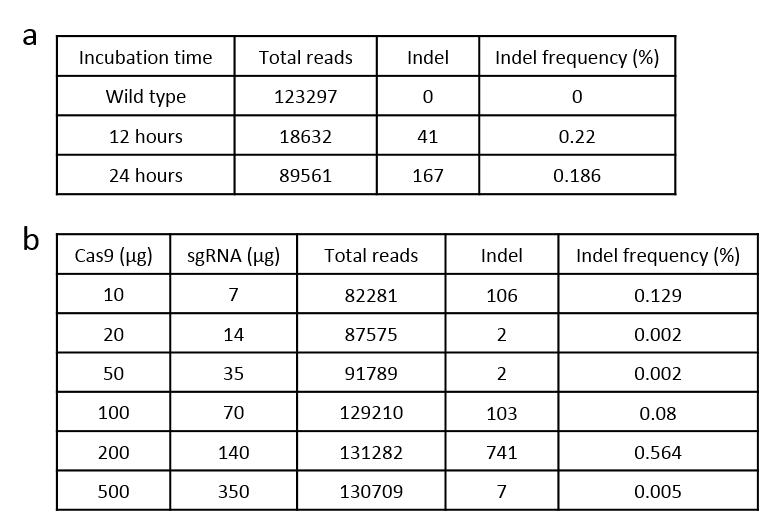
**

**Supplementary Tables
Supplementary Table 1. Quantification of pigment content, total Chl and Chl *a* to Chl *b* ratios of WT, Δ*ZEP* mutant and the Δ*ZEP*/Δ*CpFTSY* mutant lines.** Cells were grown TAP media under low light (70 μmol photons m^-2^s^-1^) conditions. Vio, violaxanthin; An, antheraxanthin; Zea, zeaxanthin. Data are the average and SE from four replicates.

|  | **Vio**  **(fmol/cell)** | **An**  **(fmol/cell)** | **Zea**  **(fmol/cell)** | **Chl *a*/*b* ratio**  **(fmol:fmol)** | **Total Chl**  **(fmol/cell)** |
| --- | --- | --- | --- | --- | --- |
| **WT** | 0.2143 ± 0.0036 | 0.0046 ± 0.0002 | 0.0074 ± 0.0004 | 2.59 ± 0.15 | 2.29 ± 0.08 |
| ***ZEP* KO Δ*Z1*** | Nd | Nd | 0.2190 ± 0.0022 | 2.53 ± 0.12 | 1.80 ± 0.04 |
| **Double KO Δ*ZF1*** | Nd | Nd | 0.1011 ± 0.0013 | 6.66 ± 0.45 | 0.49 ± 0.03 |
| **Double KO Δ*ZF2*** | Nd | Nd | 0.1314 ± 0.0019 | 5.35 ± 0.62 | 0.64 ± 0.12 |
| **Double KO Δ*ZF3*** | Nd | Nd | 0.1073 ± 0.0019 | 6.61 ± 0.03 | 0.46 ± 0.11 |
| **Double KO Δ*ZF4*** | Nd | Nd | 0.1110 ± 0.0014 | 6.94 ± 0.39 | 0.46 ± 0.13 |
| **Double KO Δ*ZF5*** | Nd | Nd | 0.1006 ± 0.0011 | 5.91 ± 0.01 | 0.56 ± 0.01 |
| **Double KO Δ*ZF6*** | Nd | Nd | 0.0923 ± 0.0012 | 6.52 ± 0.35 | 0.55 ± 0.03 |
| **Double KO Δ*ZF7*** | Nd | Nd | 0.1125 ± 0.0019 | 5.04 ± 1.15 | 0.55 ± 0.21 |
| **Double KO Δ*ZF8*** | Nd | Nd | 0.0860. ± 0.0019 | 5.90 ± 0.49 | 0.43 ± 0.11 |
| **Double KO Δ*ZF9*** | Nd | Nd | 0.0956 ± 0.0011 | 4.78 ± 1.29 | 0.52 ± 0.22 |
| **Double KO Δ*ZF10*** | Nd | Nd | 0.1128 ± 0.0005 | 6.90 ± 0.57 | 0.52 ± 0.04 |
| **Double KO Δ*ZF11*** | Nd | Nd | 0.1189 ± 0.0027 | 5.72 ± 0.07 | 0.51 ± 0.18 |
| **Double KO Δ*ZF12*** | Nd | Nd | 0.1117 ± 0.0019 | 6.18 ± 0.01 | 0.48 ± 0.18 |
| **Double KO Δ*ZF13*** | Nd | Nd | 0.0827 ± 0.0004 | 6.14 ± 0.17 | 0.54 ± 0.00 |
| **Double KO Δ*ZF14*** | Nd | Nd | 0.1215 ± 0.0021 | 6.24 ± 0.09 | 0.56 ± 0.12 |

**Supplementary Table 2. Primers used in this study**

| Name | Target site (5’ to 3’)* | Usage |
| --- | --- | --- |
| FTSY_3_1st_for | ttgcacacaagaacgcatga | PCR target locus of *CpFTSY* gene |
| FTSY_3_1st_rev | ggcattggagtaaacgaccc |
| FTSY_3_2nd_for | cgtcacctcgaatcacacac |
| FTSY_3_2nd_rev | actaacacacccacacccac |
| ZEP_3_1st_for | ctacgtcgccttactgtgtg | PCR target locus of *ZEP* gene |
| ZEP_3_1st_rev | gattgcctactcaccactcg |
| ZEP_3_2nd_for | ccaggttcaggtctttgagc |
| ZEP_3_2nd_rev | caacgctggctaaacatgct |
